# Hosting ICASA 2021 in South Africa amidst the global Omicron scare

**DOI:** 10.11604/pamj.2022.41.91.33535

**Published:** 2022-02-02

**Authors:** Mosa Moshabela, Nokukhanya Msomi, Ginette Claude Mireille Kalla, Gloria Maimela, Jean-Cyr Yombi, Francois-Xavier Mbopi-Keou

**Affiliations:** 1 University of KwaZulu Natal, Durban, South Africa,; 2 University of Yaoundé I, Faculty of Medicine and Biomedical Sciences, Yaoundé, Cameroon,; 3 The Institute for the Development of Africa (The-IDA), Yaoundé, Cameroon,; 4 University of Witwatersrand, Johannesburg, South Africa,; 5 Cliniques Universitaires Saint Luc, Département de Médecine Interne, Université Catholique de Louvain, Louvain-la-Neuve, Belgique

**Keywords:** HIV, AIDS, sexually transmitted infections, Africa, ICASA, Durban, South Africa

## Abstract

The 21^st^ International Conference on HIV/AIDS and STI's in Africa (ICASA) was successfully held from the 6^th^ to 11^t^^h^ December 2021 in Durban, South Africa. Little did we know at the time of planning that COVID-19 could become such a formidable force in eroding the progress made to bring lifesaving therapies among vulnerable communities in Africa. The conference also highlighted Africa's openness to the world, also shown in the way South Africa shared data on its discovery of the Omicron variant. Arguably the most important of lessons is that integrated HIV/TB services have become a platform on which to provide other services. We also saw how HIV and TB services were used as leverage for COVID-19 services. Much was also discussed about the need to adopt more self-care approaches, as was demonstrated with the increased use of self-testing technologies for HIV, and potentially other health needs. It's clear that Africa needs to increase its capacity to support and enable innovation, particularly in the design and manufacturing of new technologies including diagnostics, vaccines and therapeutics.

## Opinion

The 21^st^ International Conference on HIV/AIDS and STI´s in Africa (ICASA) was successfully held from the 6^th^ to 11^th^ December 2021, under the theme of Africa´s AIDS Response: the race to 2030-evidence, scale up, accelerate. The UNAIDS 90-90-90 targets were conceived with some consideration that these could be achieved by 2020. Hence these goals were re-imagined as 95-95-95 targets to be achieved by 2030, promoting the theme of the conference to be formulated as the race to 2030 in Africa´s response to HIV/AIDS. In his opening address, Deputy President David Mabuza reaffirmed South Africa´s commitment to greater unity of the African continent and further support the Common African Position developed under the African Union. Plenary speakers included H.E. Marisol Touraine, former Minister Health in France and Board Chair of UNITAID, Dr John Nkengasong, Director of Africa CDC, Dr Matshidiso Moeti, Regional Director for Africa World Health Organization and Dr Shannon Hader, Deputy Executive Director of Programme at UNAIDS and Assistant Secretary General at the United Nations. The goal of the conference was to see how we could respond to HIV in relation to related diseases such as Hepatitis, Malaria, TB, and emerging infections to achieve the following objectives: 1) strengthen health systems to integrate high impact interventions on comorbidities, emerging infections and NCDs; 2) build capacity to manufacture vaccines, diagnostics and therapeutics; 3) address response to HIV to women, children and key populations; 4) assess impact of COVID-19 and how to maintain the continuum of care; 5) integrate national and international response to curb the spread of COVID-19.

Little did we know at the time of planning that COVID-19 could become such a formidable force in eroding the progress made to bring lifesaving therapies among vulnerable communities in Africa. As the Scientific Chair of the conference Prof Francois-Xavier Mbopi-Keou was asked, “why are you going to South Africa when everyone is avoiding the country, when you should be going to Europe?” In response he said, “but there are people living there in South Africa. How are they managing?” It was soon to emerge that the virus was already in Europe weeks before ICASA took place in South Africa. Botswana reported that European delegates were among those who were sampled for the Omicron, and for all it's worth, the variant may have emerged in Europe. Whilst, COVID-19 remained a threat to the conference itself, but more so the emergence of the Omicron variant, the conference was able to go ahead.

The conference turned quickly from a planned hybrid approach to a full virtual conference as a result of increasing number of asymptomatic cases identified during screening with rapid antigen tests. There was a large number of people who had already travelled to South Africa, and was able to join virtually. One of the important lessons from this conference is future conference have to be planned with all options on the table, so that a rapid turnaround plan can be put in place. Some people drop out due to technical connectivity and other personal disruptions, and therefore backup planning appears as an important part of the process. Africa needs better connectivity, with regular interpreters on the conference platforms. The conference also highlighted Africa's openness to the world, also shown in the way South Africa shared data on its discovery of the Omicron variant, but received a backlash.

Many of the sessions were also chaired by a number of prominent African scientists who currently live and work in the global North, in the Diaspora, and are always very keen to make a contribution in addressing problems on the continent. It's imperative that we continue to engage them, and find better ways of drawing on their support at any point in time, but more so during times of crises [1].

Scientifically, a number of preliminary lessons were observed during the conference: 1) The COVID-19 pandemic has caused a lot of disruptions to HIV and TB service delivery, and in many ways, slowed the progress and momentum already gained prior to the pandemic, and this can become a major setback for sub-Saharan Africa if we are not careful and creative enough. We have indeed also observed examples of innovative solutions introduced during the conference in order to counter the erosive force of COVID-19 against HIV and TB programs, including decentralization, differentiation and integration of services, as well as provision of more holistic and personalized services. The many lessons gained in this regard should not be lost as we move forward in our path towards a new normal, that of learning to live with COVID-19, as well as a period of recovery beyond the pandemic. 2) Arguably the most important of lessons is that integrated HIV/TB services have become a platform on which to provide other services, for example family planning services, where we saw evidence of increased uptake of contraception in a pilot study from Zimbabwe. We saw how these services were used as a platform to detect mental health problems, and to introduce services for mental health. Depression was found to be an independent risk factor for non-adherence to HIV treatment. It's evident from the conference that the task of attending to the mental health and wellbeing of people living with HIV is ever so important, and those opportunities provided at every visit and interaction between health care workers (HCWs) and people leaving with HIV (PLHIV) should not become missed opportunities for the provision of a holistic package of care, including family planning services and mental health care. 3) We also saw how HIV and TB services were used as leverage for COVID-19 services, for example a pivot from typical HIV services to a combined service point with COVID-19 vaccination, as a way to increase accessibility and uptake of COVID-19 [2]. Given the nature of the relationships built between PLHIV and their service providers, we saw interventions that were able to leverage this trust relationship in order to provide linkage to care for COVID-19 awareness, prevention and vaccination. Dr John Nkengasong was able to paint a clear picture of COVID-19 in Africa, and the importance of a response to the pandemic that is African, and suitable to our very own context. Part of this context, does indeed include a high burden of HIV, TB and sexually transmitted infections (STIs), as we are all very aware. 4) Perhaps the biggest battle that we still face pertains to the efforts to identify barriers to effective HIV care for key populations, including LGBTQI+ community and sex workers. Stigma still remains one of the most challenging barriers, and this has rendered even preexposure prophylaxis (PREP) very difficult to access for the key populations. We saw some studies that were attempting to provide incentives in order to increase the uptake of PREP, but many challenges still exist in this regard. PREP is still only accessible in small number of countries in Africa, and even in those countries, there is still a problem of unequal distribution, in that some areas appear to benefit more than others. There is an urgent need for access to PREP in sub-Saharan Africa, particularly for adolescent girls and young women. The new PREP modalities that are already on the horizon, as was discussed during the conference, will need to find us already prepared with increased access, as well as expanded opportunities for choice and preference.

Much was also discussed about the need to adopt more self-care approaches, as was demonstrated with the increased use of self-testing technologies for HIV [3], and potentially other health needs. The conference demonstrated some examples of self-led contraception interventions, and also peer-led models of care, indicating the growing need for our policies to accommodate self-care and self-management. It's clear that Africa needs to increase its capacity to support and enable innovation, particularly in the design and manufacturing of new technologies including diagnostics, vaccines and therapeutics, as clearly demonstrated by H.E. Marisol Touraine during her keynote address entitled “Political leadership and partnerships to promote and accelerate access to innovation prevention and care”. Only through manufacturing can these technologies needed to fight pandemics be made easily accessible and affordable to countries in Africa. This point was also reiterated in relation to COVID-19 vaccine production, as a way to overcome the global inequities, and ensure access to vaccines on the continent.
